# WNT/β-catenin regulatory roles on PD-(L)1 and immunotherapy responses

**DOI:** 10.1007/s10238-023-01274-z

**Published:** 2024-01-27

**Authors:** Keywan Mortezaee

**Affiliations:** Department of Anatomy, School of Medicine, Kurdistan University of Medical Sciences, Sanandaj, Iran

**Keywords:** β-catenin, Immune checkpoint inhibitor (ICI), Programmed death-1 (PD-1), Programmed death-ligand 1 (PD-L1), Tumor microenvironment (TME), Resistance

## Abstract

Dysregulation of WNT/β-catenin is a hallmark of many cancer types and a key mediator of metastasis in solid tumors. Overactive β-catenin signaling hampers dendritic cell (DC) recruitment, promotes CD8^+^ T cell exclusion and increases the population of regulatory T cells (Tregs). The activity of WNT/β-catenin also induces the expression of programmed death-ligand 1 (PD-L1) on tumor cells and promotes programmed death-1 (PD-1) upregulation. Increased activity of WNT/β-catenin signaling after anti-PD-1 therapy is indicative of a possible implication of this signaling in bypassing immune checkpoint inhibitor (ICI) therapy. This review is aimed at giving a comprehensive overview of the WNT/β-catenin regulatory roles on PD-1/PD-L1 axis in tumor immune ecosystem, discussing about key mechanistic events contributed to the WNT/β-catenin-mediated bypass of ICI therapy, and representing inhibitors of this signaling as promising combinatory regimen to go with anti-PD-(L)1 in cancer immunotherapy. Ideas presented in this review imply the synergistic efficacy of such combination therapy in rendering durable anti-tumor immunity.

## Introduction

Wingless-related integration site (WNT)/β-catenin is an immunosuppressive signaling [1] that its activity in a tumor is indicative of low rates of immune infiltration [2]. WNT/β-catenin signaling is a critical mediator of melanoma metastasis [3], orchestrating a T cell exclusion profile [4]. Disruption of WNT/β‐catenin signaling is reported as a strategy for suppression of invasion and metastasis in non-small cell lung cancer (NSCLC) [5]. β-catenin activation shapes the immune desert landscape of hepatocellular carcinoma (HCC) [6]. The suppressive effect of WNT/β-catenin on CCL4 contributed to the cold immune phenotype of melanoma [7]. Mutations in the WNT/β-catenin occur in about 70% of microsatellite stable colorectal cancer (CRC) patients [8]. The frequency of β-catenin^+^ tumor cells and programmed death-ligand 1 (PD-L1)^+^ immune cells can be regarded as an indicator of CRC progression [9]. WNT/β-catenin activity promotes CRC progression through induction of epithelial-mesenchymal transition (EMT) [10]. Expression of Frizzled-10 (Fzd-10) receptor and further β-catenin activation promote cancer stem cell (CSC) expansion and predicts weak prognosis in HCC [11]. Enriched activity of this signaling in tumors with cold immunity (non-T cell-inflamed) provides a rationale for development of inhibitors in order to restore immune infiltration and increasing the efficacy of immunotherapy [12]. There are signs of evidence indicating the combination of impact of WNT/β-catenin blockade with immune checkpoint inhibitors (ICIs) for better promotion of anti-tumor immunity against cancers like NSCLC [13] and melanoma [14]. The aim of this review is to justify the mechanistic backbone of WNT/β-catenin-mediated ICI resistance, as well as rationalizing a possibility of the application of WNT/β-catenin blockade as a combinatory regimen with anti-PD-(L)1 aiming at a durable anti-cancer therapy.

## WNT/β-catenin

### Signaling elements

**Fig. 1:**
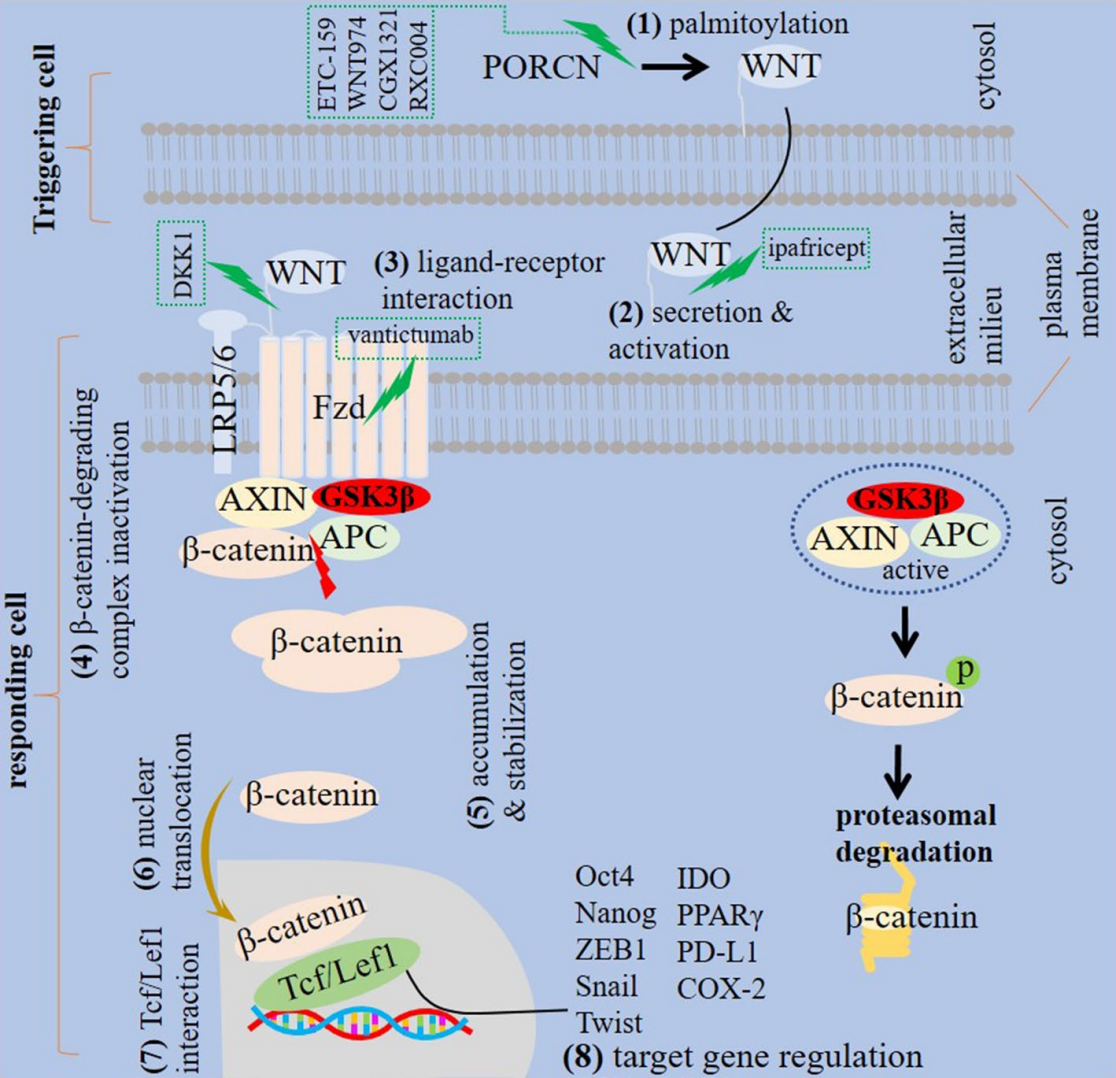
WNT/β-catenin signaling. Different steps are involved in the activity of WNT/β-catenin signaling. First, WNT palmitoylation occurs under the impact of porcupine (PORCN), which causes WNT secretion and activation. The active WNT interacts with Frizzled (Fzd)/lipoprotein receptor related proteins 5 and 6 (LRP5/6) complex in target cell and subsequently causes inactivation of β-catenin degrading complex and the resultant β-catenin cytosolic accumulation and its stabilization. The stabilized β-catenin translocate into the nucleus where it bonds to the T cell transcription factor (Tcf)/lymphoid enhancer-binding factor 1 (Lef1) for regulation of target genes. β-catenin signaling is inactivated when glycogen synthase kinase 3β (GSK3β) and the inhibitory complex is active, which subsequently promotes β-catenin proteasomal degradation. APC, adenomatosis polyposis coli; ZEB, Zinc finger E-box binding homeobox; IDO, indoleamine 2,3-dioxygenase; PPARγ, peroxisome proliferator-activated receptor-γ; and PD-L1, programmed death-ligand 1. Inhibitors of different paths in this signaling are marked as dashed rectangles

*WNT* (*WNT5a)* is a gene assessed to evaluate mesenchymal transition [15], and β-catenin is a critical mediator of WNT signaling [16]. In fact, WNT proteins co-express to act synergistically for activation of β-catenin signaling in several cell types [17]. Adenomatosis polyposis coli, CTNNB1 and AXIN (AXIN1 and AXIN2) are β-catenin signaling elements [12]. *Adenomatosis polyposis coli* is a gene related to the suppression of WNT/β-catenin [18], which shows mutations in more than 90% of sporadic colon cancer cases [19]. *CTNNB1* is a gene encoding β-catenin that is mutated frequently in HCC. *CTNNB1* mutation results in the cytoplasmic accumulation of β-catenin, which subsequently causes aberrant activation of WNT [20]. AXIN is a cytoplasmic protein that acts as a negative regulator of WNT pathway and promotes β-catenin degradation [21]. AXIN2 can be assessed as a marker for analyzing the activity of WNT pathway [22]. WNT/β-catenin pathway signals via interaction with Fzd receptor family as well as different co-receptors [23]. Low-density lipoprotein receptor related proteins 5 and 6 (LRP5/6) is a co-receptor located on cell surface that is involved in the initiation of WNT/β-catenin pathway [18]. Upon WNT activation, β-catenin degrading complex is inactivated, which results in β-catenin accumulation within the cytosol and its further stabilization. The stabilized β-catenin further translocated into the nucleus where it bonds to the T cell transcription factor (Tcf)/lymphoid enhancer-binding factor 1 (Lef1) [24, 25]. β-catenin is a Tcf1 transcriptional coactivator in which interactions within the β-catenin/Tcf1 axis are vital for transcriptional regulation. The Tcf1 long isoform contains β-catenin binding domain that mediates β-catenin recruitment to the protein complex [26]. WNT is palmitoylated by porcupine (PORCN). PORCN activity is vital for secretion of WNT and its bondage to Fzd in responder cells [27]. PORCN inhibition disrupts secretion of WNT and hampers stem cell activity in tumors [27], so it can be a target in WNT-driven cancers [28]. Fzd receptors are other targets for WNT pathway suppression in human cancers [23]. Dickkopf-related protein 1 (DKK1) is a known antagonist of WNT that acts through suppression of WNT interaction with Fzd receptors [29]. Hindering the secretion of WNT ligands, interfering with interaction between WNT ligand and receptor, increasing the degradation of β-catenin or blocking interaction between β-catenin with its target genes are strategies for hampering WNT/β-catenin signaling. Monoclonal antibodies against Fzd receptors, such as vantictumab (OMP-18R5), Fzd8 fusion proteins and extracellular traps for WNT ligand signaling, such as ipafricept (OMP-54F28), and PORCN inhibitors, such as ETC-159, LGK974 (WNT974), CGX1321 and RXC004 are targeted inhibitors of WNT/β-catenin signaling [30] (Fig. 1).

### WNT/β-catenin signaling in health and disease

WNT/β-catenin is an evolutionally conserved singling [31] that is important in establishing and maintenance of cell-to-cell adhesion [2]. Dysregulation of WNT/β-catenin accounts for diseases like cancer. When WNT ligand is not present in the environment, β-catenin is assembled in related complex and low level of β-catenin is maintained within cytosol. β-catenin further undergoes phosphorylation and degradation. By contrast, bondage between WNT with related receptors prevent β-catenin degradation and allows its accumulation within cytosol and further translocation into nucleus for activating WNT-related transcription program [32]. WNT/β-catenin maintains stemness in several epithelial tissues, which is important for development and regeneration of body organs [27]. WNT/β-catenin signaling promotes self-renewal potential of hematopoietic stem cells [31], and its sustained activity in epidermal region expands stem cell compartment in the underlying dermis [33]. Survival of immature CD4^+^ CD8^+^ T cells in thymus is also associated with β-catenin [34]. The impact of WNT (WNT3a) on self-renewal maintenance of CD8^+^ T cells, as occurring under normal conditions, represents implications of this signaling in vaccination or adoptive T cell therapy [31].

Increased β-catenin activity is a tumor hallmark [35], which is contributed to the initiation, progression, and invasion and metastasis of cancer [36]. Hyperactive WNT/β-catenin signaling promotes aberrant cellular growth during cancer initiation [18]. WNT/β-catenin is active in areas with vascular endothelial growth factor (VEGF)-related cold immunity [37], and the impact of β-catenin on P-glycoprotein is indicative of its involvement in multi-drug resistance [38, 39]. WNT/β-catenin reduces the expression of epithelial-related markers, such as E-cadherin [40], which is for acquisition of cancer stemness features. CSCs are PORCN^+^ and provide WNT within their niches for tumor progressive purposes [41].

## WNT/β-catenin impact on cellular immunity

**Fig. 2:**
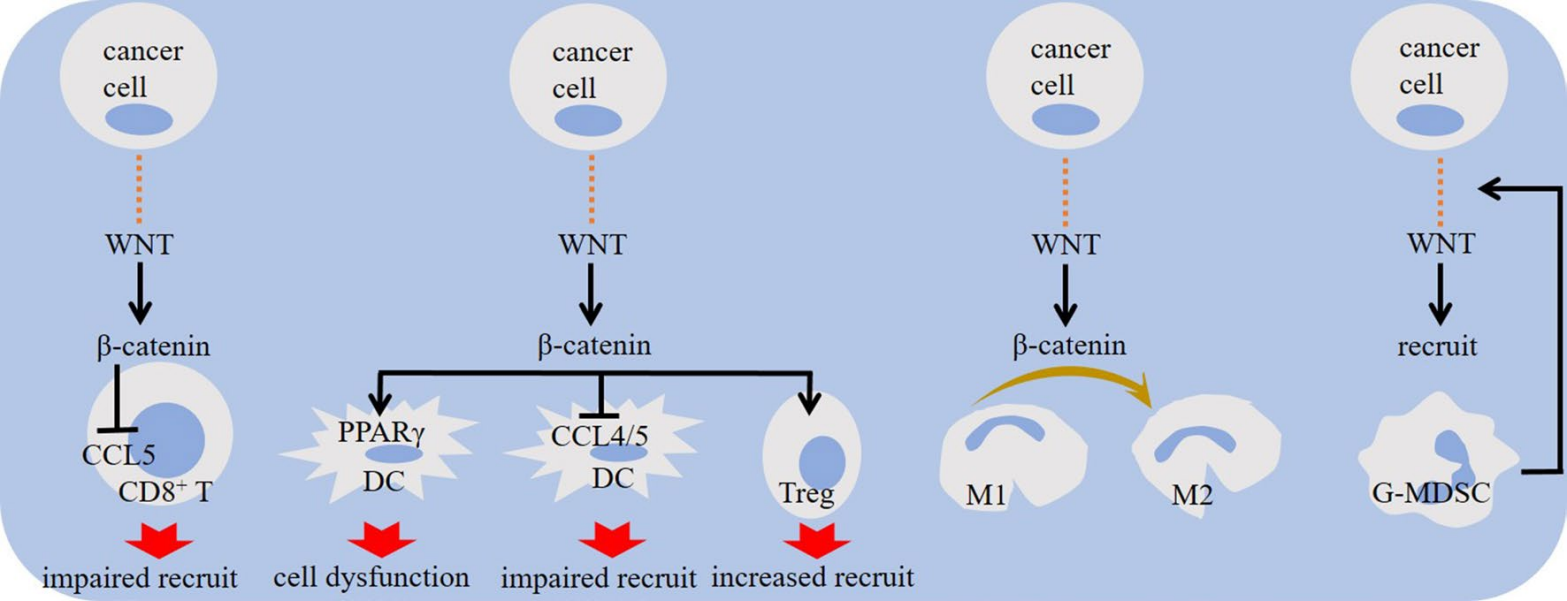
The impact of WNT/β-catenin signaling on immune cells within tumor microenvironment (TME). β-catenin activation downregulates chemokine (C–C motif) ligand 5 (CCL5) activity, re-expression of which restores immune surveillance. Defective CD8^+^ T cell priming, impaired recruitment of dendritic cells (DCs) and CD8^+^ T cells, and increased recruitment of regulatory T cells (Tregs) and granulocytic-myeloid-derived suppressor cells (G-MDSCs) are outcomes of elevated WNT/β-catenin signaling in cancer. Shifting macrophage reprogramming into pro-tumor type 2 (M2) phenotype is another outcome, which is contributed to the intensification of immunosuppressive tumor profile. PPARγ, peroxisome proliferator-activated receptor-γ

**Fig. 3:**
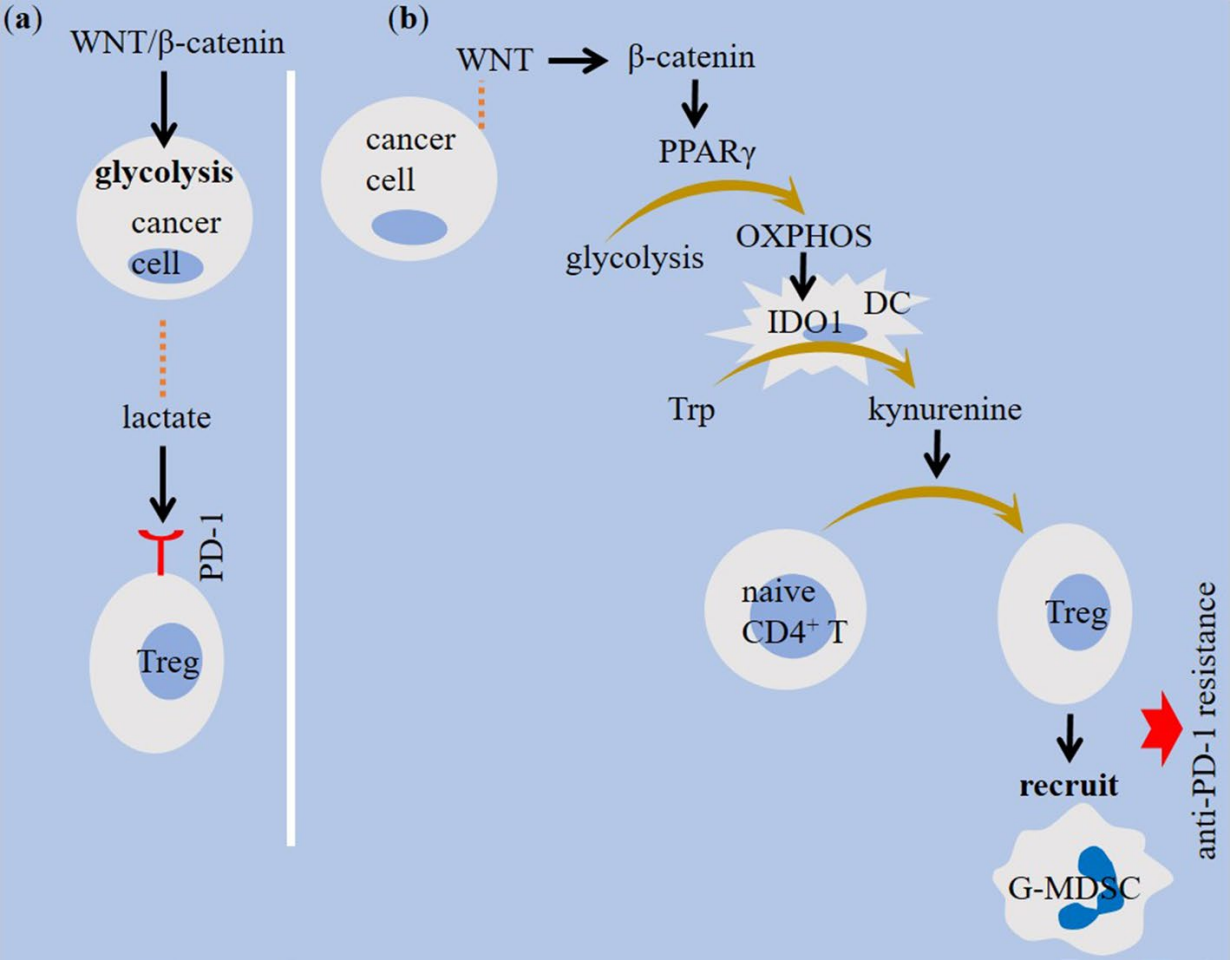
WNT/β-catenin signaling in tumor metabolism. A highly glycolytic tumor microenvironment (TME) represents high lactate release, which further acts for expression of programmed death-1 (PD-1) on regulatory T cells (Tregs). WNT5a/β-catenin induces indoleamine 2,3-dioxygenase (IDO)1 in tumor-associated dendritic cells (DCs) through activating peroxisome proliferator-activated receptor-γ (PPARγ). PPARγ reprograms DC metabolism toward oxidative phosphorylation (OXPHOS), which further increases IDO1 activity in DCs. IDO1 catalyzes tryptophan degradation, and the resultant kynurenine accumulation promotes Treg activity

Β-catenin activity mediates cooperation between tumor and stroma to promote cancer growth [42]. Primary tumors show elevated expression of WNT/β-catenin in CD8^+^ T cells. Increased expression of genes related to the WNT/β-catenin pathway in lymphocytes is contributed to the apoptosis of mature T cells, and increased β-catenin signaling in tumor cells promotes T cell exhaustion [43]. Besides, infiltration of effector CD8^+^ T cells into the tumor area is diminished under WNT/β-catenin pathway activity [30, 44]. Mutation of adenomatosis polyposis coli is contributed to the elevated β-catenin activity and reduced CD8^+^ T cell proportion in the TME of CRC [45]. There is a strong correlation between regulatory T (Treg) intra-tumoral recruitment with mutation of adenomatosis polyposis coli in CRC [46]. β-catenin acts on Tcf/Lef, which are transcription factors important for promoting immunosuppressive activity of Tregs [47]. β-catenin also reduces levels of chemokines contributed to the recruitment of dendritic cells (DCs) into tumor area [48, 49]. β-catenin downregulates CCL5 Chemokine (C–C motif) ligand 5 (CCL5) [49], which is involved in T cell [50] and DC [51] recruitment. WNT/β-catenin also promotes DC tolerance [30]. Hampering CD103^+^ DC recruitment by tumor cell-intrinsic WNT/β-catenin results in defective CD8^+^ T cell priming [44] (Fig. 2). Increased activity of WNT5a/β-catenin is contributed to the indoleamine 2,3-dioxygenase 1 (IDO1) induction in tumor-associated DCs [30], which is seemingly mediated through peroxisome proliferator-activated receptor-γ (PPARγ) activation [52] and further reprogramming of DC metabolism from glycolysis into oxidative phosphorylation [14] (Fig. 3). Granulocytic-myeloid-derived suppressor cell (G-MDSC) is another cell type highly expressing canonical WNT [53]. WNT signaling promotes G-MDSC recruitment into tumor area [30], and the activity of WNT in G-MDSCs is for the subsequent induction of aberrant WNT/β-catenin activation in malignant cells for promoting breast cancer metastasis [53]. Finally, tumor-associated macrophages (TAMs) are cells upregulating WNT/β-catenin [54]. WNT ligands derived from tumor cells promote macrophage type 2 (M2) polarization through canonical pathway [55]. β-catenin ablation in TAMs by approaches like CD200R1-Ig expressing adenoviral therapy suppresses M2 polarity [56]. β-catenin blockade may even promote a M2-to-M1 shift in macrophages [54] (Fig. 2).

## WNT/β-catenin regulatory roles on PD-1/PD-L1 and ICI responses

Increased PD-L1 expression is placed downstream to the β-catenin activity [38]. Bondage of β-catenin/Tcf/Lef complex to the promoter of *CD274* gene induces PD-L1 expression on tumor cells [57], and the impact of WNT/β-catenin on PD-L1 activation is indicative of the key role of this signaling in regulation of tumor immune landscape [58]. Increased activity of WNT/β-catenin signaling is the underlying mechanism contributed to the development of non-inflamed TME and low ICI responses in highly mutated cancer type like NSCLC. In such cancer type, high tumor-mutational burden (TMB) is representative of low responses to ICI therapy [13]. This is in contrast with the common belief that a tumor with higher somatic mutations generally shows higher responses to immunotherapy due to being more accessible to be killed by immune system [59]. The high TMB in NSCLC is accompanied by lack of CD8^+^ T cell in TME and the resultant promotion of ICI resistance. This is due to the increased activity of WNT/β-catenin, which impairs CD8^+^ T cell infiltration into the tumor area [13]. β-catenin activation is contributed to anti-PD-1 resistance in HCC [49]. B-cell lymphoma 9 (BCL9) is the co-activator of β-catenin. Pharmacological blockade of β-catenin/BCL9 using desired peptides is reported to reduce the proportion of Tregs, increased tumoral infiltration of cytotoxic T lymphocytes and sensitized cancer cells to anti-PD-1 therapy [46]. Lack of T cell genomic signature and T cell infiltrate due to the intrinsic tumor-mediated WNT/β-catenin activity is contributed to the anti-PD-L1 resistance of melanoma [4]. There is a report of increased WNT/β-catenin in CD8^+^ T cells after anti-PD-1 therapy of primary sarcomas [43]. Constitutive activation of WNT/β-catenin and further decreased expression of the chemokine CCL4 seemingly account for ineffective ICI responses [60]. WNT/β-catenin mediates resistance to ICI therapy in part through blockade of cytokines contributed to the recruitment of immune cells. Targeting CTNNB1 using the nanoparticle drug product DCR-BCAT is attested to augmented T cell infiltration and increased tumor sensitivity to ICI therapy [61].

**Fig. 4:**
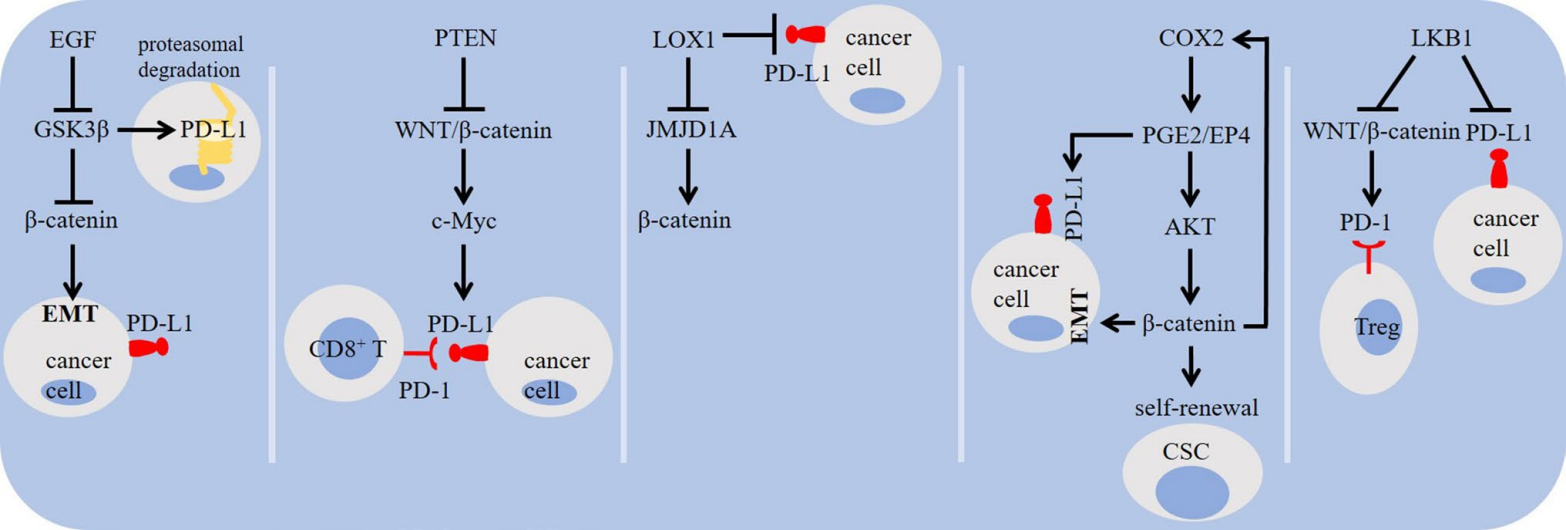
Signaling pathways related to the WNT/β-catenin activity and checkpoint regulation in cancer. Epidermal growth factor (EGF) inhibits glycogen synthase kinase 3β (GSK3β), induces β-catenin, and stimulates programmed death-ligand 1 (PD-L1) glycosylation. Activation of GSK3β destabilizes PD-L1 through promoting its ubiquitination and proteasomal degradation. β-catenin activity increases c-Myc, the activity of which enforces PD-L1 expression in tumor microenvironment (TME) and the subsequent apoptosis of T cells. The histone demethylase inhibitor 5-carboxy-8-hydroxyquinoline (IOX1) suppresses Jumonji domain-containing 1A (JMJD1A) and its downstream β-catenin, and downregulates PD-L1 on tumor cells. Prostaglandin E2 (PGE2) stimulates the activity of β-catenin for maintaining cancer stemness. PGE2 release from M2 macrophages also induces PD-L1 expression on tumor cells. Promoter of cyclooxygenase-2 (COX-2) contains Tcf4 binding element to which β-catenin is bonded for upregulation of COX-2 expression. PTEN, phosphatase and tensin homolog deleted on chromosome 10; and LKB1, liver kinase B1

**Fig. 5:**
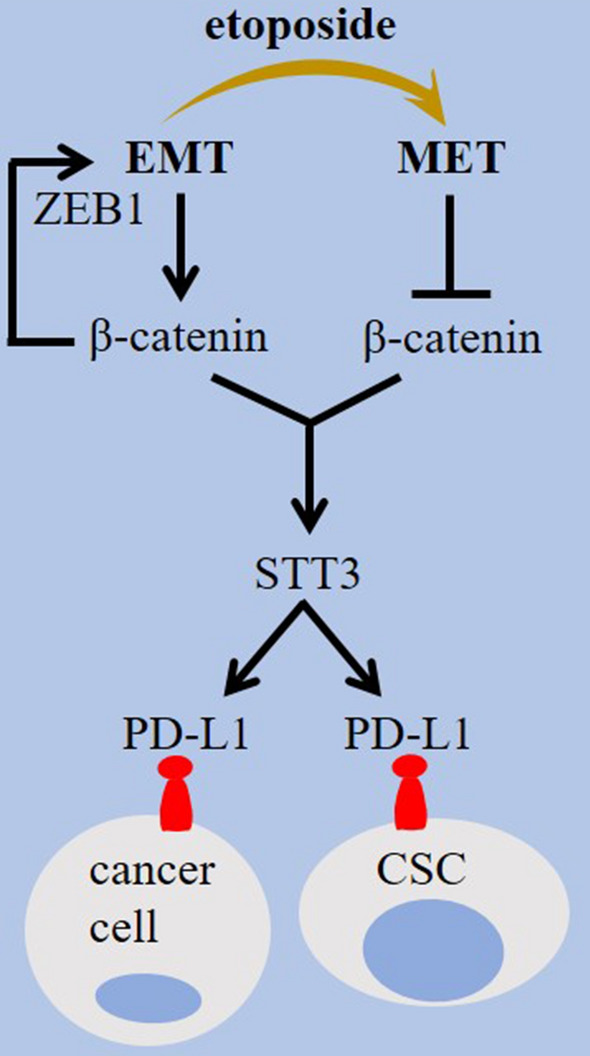
Epithelial mesenchymal plasticity in β-catenin and checkpoint regulation. Zinc finger E-box binding homeobox1 (ZEB1) is an epithelial-mesenchymal transition (EMT)-related transcription factor that its expression is induced by the β-catenin/Tcf4 complex. The N-glycosyltransferase STT3 is stimulated by EMT inducible effect on β-catenin in cancer cells and cancer stem cells (CSCs) to promote programmed death-ligand 1 (PD-L1) upregulation. Conversion into mesenchymal–epithelial transition (MET) phenotype reduces nuclear β-catenin, downregulates PD-L1, and sensitizes tumor cells to immunotherapy

The activity of WNT/β-catenin is hampered by glycogen synthase kinase 3β (GSK3β) [31, 62, 63]. β-catenin is a GSK3β substrate [64]. GSK3β acts for promoting PD-L1 ubiquitination and degradation [65–67] (Fig. 1). GSK3β inhibition, β-catenin induction and PD-L1 glycosylation are mediated under the influence of epidermal growth factor (EGF) [68], and that GSK3β activators can be used for PD-L1 instability and increasing anti-PD-1 efficacy [65]. WNT/β-catenin stimulates glycolysis [69], and the highly glycolytic TME shapes the immune landscape of tumor through inducing the expression of PD-1 on Tregs [70] (Fig. 3). WNT/β-catenin induces PD-L1 transcription and T cell apoptosis through stimulating c-Myc signaling in hepatitis B virus (HBV) mouse model and HBV^+^ hepatoma cells, which is counteracted by phosphatase and tensin homolog deleted on chromosome 10 (PTEN) [71]. Trujillo and colleagues described mechanistic backbone of resistance to the combined anti-PD-1 and anti-CTLA-4 in two cases of metastatic melanoma and noticed a robust tumoral expression of β-catenin in a one and acquired PTEN loss in another, with both evolving loss of T cell infiltration [72]. β-catenin also cooperate with prostaglandin E2 (PGE2) in cancer [73], and the release of PGE2 from M2 TAMs induces PD-L1 on tumor cells [74]. Study shows a possible correlation between PGE2 generation and increased β-catenin activity for maintaining stemness in glioblastoma tumor cells [75]. Promoter of cyclooxygenase-2 (COX-2) contains Tcf4 binding element to which β-catenin is bonded for further upregulation of COX-2 in colon and liver cancer [76]. β-catenin also interacts with liver kinase B1 (LKB1) to control PD-1 activity [16]. Silencing intracellular LKB1 is also followed by an increase in the level of PD-L1 [77], and the loss of *Stk*11/*Lkb*1 is reported to promote resistance to anti-PD-(L)1 in KRAS mutant lung adenocarcinoma [78] (Fig. 4). Finally, β-catenin/Tcf4 induces Zinc finger E-box binding homeobox1 (ZEB1), a known mediator of EMT [79], and that EMT induction is linked positively with PD-L1 expression on tumor cells, as evidenced by the implication of the EMT activator ZEB1 in relieving miR-200-mediated repression of PD-L1 activity on tumor cells [80]. Etoposide is a chemotherapy drug that mediates mesenchymal–epithelial transition (MET) to reduce nuclear β-catenin and the resultant downregulation of PD-L1 on tumor cells [81] (Fig. 5).

## Combination of WNT/β-catenin inhibitors with anti-PD-1/PD-L1

Inhibitors of WNT/β-catenin can be developed to exert synergistic anti-tumor effects with ICIs in cancer immunotherapy [82]. There is a report in HCC mice model indicating potent anti-tumor efficacy of nanoparticles constructed to simultaneously target hyperactive WNT/β-catenin and block endogenous PD-L1 [83]. Takeuchi and colleagues attested a positive impact of WNT/β-catenin on ICI resistance in TMB^high^ NSCLC, and the combination therapy with WNT/β-catenin blockade and anti-PD-1 better promoted anti-tumor immunity compared with either agent alone [13]. Microsatellite stable (MSS) CRC shows dismal responses (0%) to ICI therapy. Combination of the PORCN inhibitor ETC-159 with the PD-1 inhibitor nivolumab reduced tumor volume in mice engrafted with MSS CRC. Combination therapy increased the fraction of effector CD4^+^ and CD8^+^ T cells and reduced Treg population, and augmented the antigen presentation profile represented by increased tumoral cell expression of major histocompatibility complex class II (MHC II) [84]. Elevated activity of WNT ligand signaling is also responsible for failure of anti-PD-1 in melanoma. Suppression of WNT ligand increases the efficacy of anti-PD-1 in autochthonous animal tumor models through reduction of G-MDSC recruitment and reversion of DC tolerance. The higher suppressive impact of vantictumab or ipafricept over solo anti-PD-1 is reported in animal tumor model of melanoma, which is correlated with higher intra-tumoral infiltration of tumor-specific CD8^+^ T cells. DeVito and colleagues attested a positive link between anti-PD-1 resistance with increased WNT ligand signaling, which is indicative of the sensitivity of anti-PD-1 refractory melanoma to the WNT ligand blockade, as shown after application of ETC-159 [30]. The efficacy of WNT974 plus the PD-1 inhibitor spartalizumab was evaluated in patients with advanced solid cancers. Treatment-related adverse events (TRAEs) were reported in 78% of patients, with hypothyroidism identified in 19% of cases. 53% of patients who were refractory to prior anti-PD-1 showed stable disease, with uveal melanoma all cases (*n* = 5) represented stable disease. The outcomes are indicative of a presumable synergistic activity of the combined WNT pathway inhibition with ICI therapy against advanced solid cancers [22] (Table 1).

**Table 1:** Targeting WNT-β-catenin in cancer immunotherapy

Cancer type	Target regimen	Effects	References
NSCLC	WNT/β-catenin blockade plus anti-PD-1	Combination therapy better promoted anti-tumor immunity	[13]
MSS CRC	PORCN inhibitor ETC-159 plus anti-PD-1 (nivolumab)	Combination therapy in in mice engrafted tumor reduced tumor volume, increased the proportion of effector CD4^+^ and CD8^+^ T cells and reduced Treg population	[84]
Melanoma	ETC-159 plus anti-PD-1	Anti-PD-1 resistance is linked positively with increased WNT ligand signaling, and anti-PD-1 refractory melanoma is sensitive to the ETC-159 therapy	[30]
Advanced solid cancers	WNT974 plus anti-PD-1 (spartalizumab)	Combination therapy resulted in a stable disease in 53% of patients who were refractory to prior anti-PD-1, with uveal melanoma all cases had stable disease	[22]
HCC	Nanoparticle-based inhibition of β-catenin and PD-L1	Nanoparticle delivery increased intra-tumoral proportion and activity of CD8^+^ T cells, and it showed higher anti-tumor effects compared with anti-PD-L1 in orthotopic homograft animal model	[83]
Xenograft model	WNT inhibitors plus anti-PD-L1	WNT blockade increased anti-PD-L1 efficacy through hampering CAF-related immunotherapy resistance	[85]

MSS, microsatellite stable; CRC, colorectal cancer; PD-1, programmed death-1; Treg, regulatory T; HCC, hepatocellular carcinoma; PD-L1, programmed death-ligand 1; and CAF, cancer-associated fibroblast

In summary, it is rationale to assert that dysregulation of the WNT/β-catenin occurs in the context of human cancers and is associated with several cellular processes involved in tumor progression. Failure of anti-checkpoint therapy is a multi-mechanistic issue, among which the activity of WNT/β-catenin signaling has recently taken important consideration due to its critical association with cancer stemness. Tight interactions between WNT/β-catenin with different cells within tumor immune ecosystem, close interactions with PD-1/PD-L1 axis, and the promising outcomes from clinical trials targeting the two are all indicative of the application of combination therapies using WNT/β-catenin inhibitors with anti-PD-(L)1 in cancer immunotherapy, particularly in tumors with cold immunity and highly aggressive profile. However, there are points require attention when interpreting outcomes in patients under exposure to the combined WNT/β-catenin inhibitor/anti-PD-(L)1 therapy. First, interactions between WNT with complex receptors can activate signaling either dependent or independent on β-catenin, and a hallmark of a β-catenin-dependent pathway is its stability and nuclear translocation [86]. Second, β-catenin transactivation can also occur independent on WNT [87], and Tcf1/Lef1 can also be activated by other transcription factors, such as ATF2 [88]. Third, genotoxic agents can activate WNT/β-catenin independent on canonical Fzd/LRP receptor complex [89]. Further studies are demanded for surveying other upstream mediators or inhibitors of β-catenin activity. 5-carboxy-8-hydroxyquinoline (IOX1), for instance, is a histone demethylase inhibitor that suppresses Jumonji domain-containing 1A (JMJD1A) and its downstream β-catenin, and downregulates tumoral PD-L1, expressed secondary to the doxorubicin chemotherapy [38] (Fig. 4). The presence of WNT/β-catenin signaling in circulating extracellular vesicles (EVs) [90], and surface representation of PD-L1 by EVs secreted from tumor cells [91] are all indicative of a possibility for application of EVs in cancer immunotherapy targeting both signaling. A key virtue of such strategy is the tendency of EVs for their preferential attraction toward tumor tissue area due to expressing receptors related to that tumor type. Besides WNT/β-catenin, the activity of TGF-β signaling is also contributed to the stemness of tumor cells and cancer resistance to ICI therapy. Bispecific antibodies against TGF-β and PD-L1 are developed, and impressive responses are for PD-L1^high^ platinum refractory NSCLC patients [92].
